# Performance validity in outpatients with multiple sclerosis and cognitive complaints

**DOI:** 10.1177/13524585211025780

**Published:** 2021-07-02

**Authors:** IM Nauta, D Bertens, M van Dam, M Huiskamp, S Driessen, JJG Geurts, BMJ Uitdehaag, L Fasotti, HE Hulst, BA de Jong, M Klein

**Affiliations:** Amsterdam UMC, Vrije Universiteit Amsterdam, Department of Neurology, MS Center Amsterdam, Amsterdam Neuroscience, Amsterdam, The Netherlands; Donders Institute for Brain, Cognition and Behaviour, Radboud University, Nijmegen, The Netherlands/Klimmendaal Rehabilitation Center, Arnhem, The Netherlands; Amsterdam UMC, Vrije Universiteit Amsterdam, Department of Anatomy and Neurosciences, MS Center Amsterdam, Amsterdam Neuroscience, Amsterdam, The Netherlands; Amsterdam UMC, Vrije Universiteit Amsterdam, Department of Anatomy and Neurosciences, MS Center Amsterdam, Amsterdam Neuroscience, Amsterdam, The Netherlands; Amsterdam UMC, Vrije Universiteit Amsterdam, Department of Medical Psychology, Amsterdam Neuroscience, Amsterdam, The Netherlands; Amsterdam UMC, Vrije Universiteit Amsterdam, Department of Anatomy and Neurosciences, MS Center Amsterdam, Amsterdam Neuroscience, Amsterdam, The Netherlands; Amsterdam UMC, Vrije Universiteit Amsterdam, Department of Neurology, MS Center Amsterdam, Amsterdam Neuroscience, Amsterdam, The Netherlands; Donders Institute for Brain, Cognition and Behaviour, Radboud University, Nijmegen, The Netherlands/Klimmendaal Rehabilitation Center, Arnhem, The Netherlands; Amsterdam UMC, Vrije Universiteit Amsterdam, Department of Anatomy and Neurosciences, MS Center Amsterdam, Amsterdam Neuroscience, Amsterdam, The Netherlands; Amsterdam UMC, Vrije Universiteit Amsterdam, Department of Neurology, MS Center Amsterdam, Amsterdam Neuroscience, Amsterdam, The Netherlands; Amsterdam UMC, Vrije Universiteit Amsterdam, Department of Medical Psychology, Amsterdam Neuroscience, Amsterdam, The Netherlands

**Keywords:** Multiple sclerosis, cognitive impairment, performance validity, suboptimal performance, neuropsychological assessment

## Abstract

**Background:**

Suboptimal performance during neuropsychological assessment renders cognitive test results invalid. However, suboptimal performance has rarely been investigated in multiple sclerosis (MS).

**Objectives:**

To investigate potential underlying mechanisms of suboptimal performance in MS.

**Methods:**

Performance validity testing, neuropsychological assessments, neuroimaging, and questionnaires were analyzed in 99 MS outpatients with cognitive complaints. Based on performance validity testing patients were classified as valid or invalid performers, and based on neuropsychological test results as cognitively impaired or preserved. Group comparisons and correlational analyses were performed on demographics, patient-reported, and disease-related outcomes.

**Results:**

Twenty percent displayed invalid performance. Invalid and valid performers did not differ regarding demographic, patient-reported, and disease-related outcomes. Disease severity of invalid and valid performers with cognitive impairment was comparable, but worse than cognitively preserved valid performers. Lower performance validity scores related to lower cognitive functioning, lower education, being male, and higher disability levels (*p* < 0.05).

**Conclusion:**

Suboptimal performance frequently occurs in patients with MS and cognitive complaints. In both clinical practice and in cognitive research, suboptimal performance should be considered in the interpretation of cognitive outcomes. Identification of factors that differentiate between suboptimal and optimal performers with cognitive impairment needs further exploration.

## Introduction

Neuropsychological assessments yield cognitive impairment in 43%–70% of patients with MS.^1^ However, if patients do not perform to the best of their abilities, neuropsychological test results are not a valid reflection of their actual cognitive status.^2,3^ The validity of cognitive test results can be assessed with performance validity tests (PVTs). These tests are usually easy to perform and relatively insensitive to neurological and cognitive impairments. Only when patients have serious cognitive impairments, such as Alzheimer’s disease, PVTs tend to be less reliable.^2,3^ Hence, low scores on PVTs are indicative of suboptimal performance, and if these scores are not considered when evaluating neuropsychological outcomes, patients may be incorrectly characterized as cognitively impaired.^2^ Importantly, PVT failure does not indicate intentionality, nor the absence of genuine cognitive impairment, but they do suggest that cognitive scores cannot be validly interpreted.^2^ Even though the use of PVTs is common practice in clinical practice,^2,4^ assessing performance validity has not been incorporated in international recommendations for cognitive monitoring in MS.^5^

The few studies that report on performance validity in MS revealed suboptimal cognitive performance in 11%–21% of patients referred for clinical neuropsychological testing^6,7^ and in 13% of patients tested in study contexts.^8^ These base rates are commensurate with those seen across other clinical samples such as patients with mild traumatic brain injury.^9^ Psychological, demographic, and disease-related factors may all contribute to suboptimal performance in MS. Depressive symptoms^6,7^ and anxiety,^7^ for instance, have been related to suboptimal performance in MS, although not in a consistent way.^8^ Psychological symptoms have been linked to suboptimal cognitive performance in other clinical populations, yet it has been argued that psychological factors on their own are not sufficient to cause poor performance on PVTs.^10^ Importantly, suboptimal performance rates seem higher among clinical populations characterized by fatigue and pain.^11^ Both these symptoms are frequently found in MS patients,^12^ but they have not yet been studied in relation to performance validity. In addition, it remains unclear whether disease-related symptoms may affect suboptimal performance during neuropsychological testing: previous studies reported that MS patients with suboptimal performance were younger at symptom onset^6^ and had higher neurological disability levels,^8^ whereas no association was found with magnetic resonance imaging (MRI) lesion load, atrophy, nor disease duration.^7^ Higher rates of suboptimal performance were found in MS patients applying for a disability allowance, which is in line with research on performance validity in the forensic field.^7^ Taken together, previous studies suggest several potential reasons for suboptimal cognitive performance in MS, but, given the limited number of studies and contradictory findings, there is no generally accepted explanation for suboptimal cognitive performance in MS.

In line with the literature,^6–8^ we regularly observe suboptimal cognitive performance in our MS outpatients with cognitive complaints. This observation and the limited literature on performance validity in MS prompted us to characterize patients who showed indications of suboptimal cognitive performance. Specifically, we aimed to determine which demographic, psychological, or disease-related outcomes were associated with suboptimal performance in MS patients, both within our total MS sample as well as within patients categorized as cognitively impaired and cognitively preserved. Knowledge on the underlying mechanisms of suboptimal cognitive performance in MS is important in clinical care to provide adequate patient education and counseling.

## Methods

### Patients

This cross-sectional study retrospectively analyzed data collected at the SOMSCOG (i.e. Second Opinion MS and COGnition) outpatient clinic of the MS Center Amsterdam since its start (February 2017) until February 2020. Patients with a diagnosis of MS or clinically isolated syndrome (CIS) visited this outpatient clinic because of cognitive complaints and were referred by a primary care physician or medical specialist. Patients were included if they gave written informed consent, performed the PVT (i.e. Amsterdam Short-Term Memory (ASTM) test, see section “Measures”),^3^ and were able to speak Dutch.

### Ethics

The Medical Ethics Research Committee of Amsterdam UMC concluded that the Medical Research Involving Human Subjects Act (WMO) did not apply to this study, as the data collection was part of clinical care (number METC-2016.395). Written informed consent was obtained from all patients.

### Measures

#### Demographics

The demographic characteristics including age, sex, work status, and level of education were collected. Education was coded according to Verhage^13^ and categorized as low (i.e. completed average-level secondary education or lower; levels 1–5) or high (i.e. completed high-level secondary education or university degree; levels 6–7).

#### Disease status

MS type, disease duration, and disease-modifying therapy (yes/no) were collected from the medical charts. Disability level was assessed by a certified examiner using the Expanded Disability Status Scale (EDSS).^14^ Patients were scanned on a 3-Tesla whole-body MRI (General Electric Signa HDxt), as described previously,^15^ and lesion load and whole-brain volume were calculated as indicators of cerebral damage. Lesion load was calculated after automatically segmenting fluid-attenuated inversion recovery (FLAIR) images. Whole-brain volume was calculated using FSL after filling 3DT1 images (using LEAP). Both volumes were normalized using the V-scaling factor (see Supplementary Information).^15^

#### Neuropsychological examination

Cognitive function was measured with a test battery based on the MACFIMS,^16^ and consisted of the following five (sub-)domains: (1) verbal memory (Dutch version of the California Verbal Learning Test version 2),^17^ (2) visuospatial memory (Brief Visuospatial Memory Test–Revised),^18^ (3) processing speed (Symbol Digit Modalities Test^19^ and Stroop Color–Word Test cards I and II),^20^ (4) executive function—verbal fluency (Controlled Oral Word Association Test),^21^ and (5) executive function—response inhibition (Stroop Color-Word Test interference score).^20^

Scores were corrected for age, education, and sex when applicable, and transformed into five (sub-)domain-specific *z*-scores as well as one composite score for overall cognitive functioning (i.e. average of all *z*-scores) based on a normative sample of healthy controls.^22,23^ Patients were classified as cognitively impaired (i.e. ⩾1.5 standard deviations (SDs) below the means of healthy controls on ⩾20% of the neuropsychological test scores, corresponding to ⩾3/11 test scores) or cognitively preserved (i.e. remainder). Supplementary Information provides more details on test scores and its transformation.

#### Performance validity

Performance validity was assessed with the ASTM, a forced-choice verbal recognition test specifically designed to indicate whether patients perform below their actual level of competence.^3^ The memory load is kept to a minimum and as each item is from a different semantic category, there is no interference from previous items (for a detailed description, see Schagen et al.^3^). This test consists of 30 items and scores range between 0 and 90. Invalid performance on the ASTM (i.e. below the cut-off score) is indicative for suboptimal performance regarding the neuropsychological assessment. The recommended cut-off score is ⩽84 (specificity = 90% and sensitivity = 84%).^24^ For this study, a higher specificity (>90%) was considered important to reduce the risk of false positives (i.e. incorrectly indicating suboptimal performance), and we therefore applied a cut-off score of ⩽82 (specificity = 95% and sensitivity = 67%).^24^

Moreover, we reported on performance validity indices within conventional neuropsychological tests (i.e. embedded PVT measures; Table 4).^25,26^ Embedded PVTs have known drawbacks, including reduced sensitivity levels relative to stand-alone PVTs,^25^ but may be informative in case stand-alone PVTs were not administered.

#### Patient-reported outcomes

The MS Neuropsychological Questionnaire patient version measured cognitive complaints.^27^ The Hospital Anxiety and Depression Scale measured symptoms of anxiety and depression.^28^ The subscale “subjective experience of fatigue” of the Checklist Individual Strength-20-r measured the level of fatigue.^29^ The Athens Insomnia Scale measured sleep disturbances.^30^ For all of the aforementioned questionnaires, higher scores indicate more symptoms. The Utrecht Coping List measured coping style (Tables 2 and 3 provide subscales).^31^ Higher scores indicate that a patient predominantly adopts a specific coping style. The MS Quality of Life Questionnaire-54 measured physical and mental quality of life, and we also focused on the pain subscale. Higher scores represent better quality of life.^32^ Supplementary Information provides more details.

### Statistical analyses

By applying the PVT cut-off score, patients’ test performance was classified as valid or invalid. For ease of survey, patients were indicated as valid or invalid performers. Within the total sample and cognitively impaired group, differences between valid and invalid performers were analyzed regarding demographic, disease-related, patient-reported, and cognitive outcomes (i.e. differences were not analyzed within the cognitively preserved group due to the small sample size of invalid performers within this subgroup (*N* = 2)). These outcomes were also compared across the following three subgroups: cognitively impaired invalid performers, cognitively impaired valid performers, and cognitively preserved valid performers. Significant effects were further analyzed to investigate which groups differed significantly. Depending on psychometric properties of outcome measures, two-group comparisons were analyzed using independent samples *t*-tests, Mann–Whitney *U* tests, chi-square tests or Fisher’s exacts, and three-group comparisons with analyses of variance, Kruskal–Wallis tests, chi-square tests, or Fisher–Freeman–Halton tests.

Within the total sample, taking the skewed distribution of the PVT into account, Spearman’s correlations were calculated between the PVT score and demographic, disease-related, patient-reported, and cognitive outcomes. In addition, a linear regression analysis was performed to determine the amount of variance in overall cognitive functioning that could be explained by the PVT score. Finally, the concordance between the stand-alone PVT (i.e. ASTM) and embedded PVTs were reported.

Significance level was set at *p* < 0.05, and Bonferroni-corrected if variables consisted of multiple subscales (0.05 divided by number of subscales; see notes in Tables 2 and 3). The statistical analyses were performed in SPSS 26.0.

## Results

**Figure 1.**
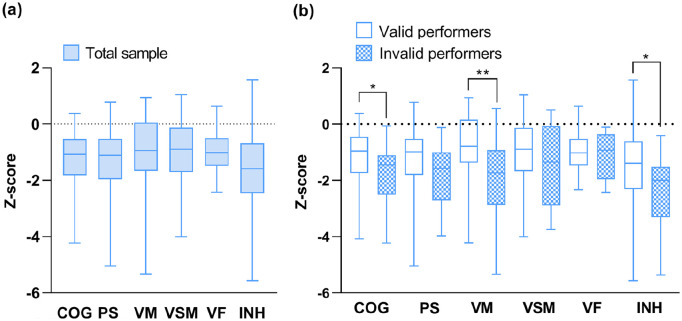
Distribution of cognitive scores in the total sample and stratified by performance validity. (a) Cognitive scores per domain of the total sample, irrespective of performance validity. (b) Cognitive differences between the valid performers and invalid performers per domain. COG: overall cognitive functioning; PS: processing speed; VM: verbal memory; VSM: visuospatial memory; VF: verbal fluency; INH: inhibition. *p < 0.01; **p < 0.001.

Based on the inclusion criteria, our final sample consisted of 99 patients (66% female, age 47.6 ± 9.6 years, symptom duration 14.8 ± 9.1 years, median EDSS = 3.5 (range = 1.5–7.5), 66% relapsing-remitting multiple sclerosis (RRMS)) out of 129 patients who had visited our outpatient clinic. Regardless of performance validity, 63% of our sample was categorized as cognitively impaired. Figure 1 presents the cognitive scores of the total sample for each cognitive (sub-)domain.

### Performance validity of the total sample

Twenty percent (20/99) of the patients scored below the PVT cut-off. These invalid performers did not differ from valid performers regarding demographic, disease-related, and patient-reported outcomes (Tables 1 and 2). Compared to valid performers, invalid performers had lower overall cognitive functioning (*p* = 0.001), verbal memory (*p* < 0.001), and response inhibition (*p* = 0.004; Figure 1; Table 2). Fifty-six percent of valid performers were classified as cognitively impaired, while this was the case in 90% of invalid performers.

**Table 1.** Demographic and disease-related characteristics stratified by performance validity.

	Total sample (*N* = 99)			Classified as cognitively impaired (*N* = 62)			Classified as cognitively preserved (*N* = 37)	
	Valid performers (*N* = 79, 80%)	Invalid performers (*N* = 20, 20%)	*p*	Valid performers (*N* = 44, 71%)	Invalid performers (*N* = 18, 29%)	*p*	Valid performers (*N* = 35, 95%)	*p* ^e^
Demographics								
Age (years), mean (SD)	47.3 (9.3)	48.9 (10.9)	0.510	47.4 (8.9)	49.6 (11.2)	0.422	47.0 (9.9)	0.641
Sex (women), *n* (%)	54 (68%)	11 (55%)	0.261	24 (55%)	10 (56%)	0.942	30 (86%)	0.009*
Education (high), *n* (%)	48 (61%)	9 (45%)	0.203	26 (59%)	9 (50%)	0.512	22 (63%)	0.665
Work status,^a^ *n* (%)			0.155			0.314		0.492
Employed (paid/unpaid)	15 (19%)	3 (15%)		7 (16%)	2 (11%)		8 (23%)	
Employed with sickness benefits (partly/fully)	11 (14%)	8 (40%)		6 (14%)	7 (39%)		5 (14%)	
Disability pension (partly/fully)	38 (48%)	8 (40%)		21 (48%)	8 (44%)		17 (49%)	
Unemployed	5 (6%)	1 (5%)		4 (9%)	1 (6%)		1 (3%)	
Other (student, homemaker, retired)	6 (8%)	0 (0%)		3 (7%)	0 (0%)		3 (9%)	
Unknown	4 (5%)	0 (0%)		3 (7%)	0 (0%)		1 (3%)	
Disease-related characteristics								
MS type (CIS/RRMS/SPMS/PPMS/unknown),^a,b^ %	5/66/19/8/3%	0/65/10/20/5%	0.227	5/59/27/7/2%	0/61/11/22/6%	0.149	6/74/9/9/3%	0.088
Disease duration (years),^c^ median (IQR)	14.8 (13.7)	12.5 (14.3)	0.159	15.8 (13.3)	13.0 (15.0)	0.242	13.9 (12.7)	0.503
EDSS, median (range)	3.5 (1.5–7.5)	4.0 (3.0–6.0)	0.194	4.0 (2.0–7.5)	4.0 (3.0–6.0)	0.943	3.0 (1.5–7.0)	0.005*
DMT use (yes), *n* (%)	41 (52%)	8 (40%)	0.342	23 (52%)	6 (33%)	0.175	18 (51%)	0.363
Lesion load (mL),^d^ median (IQR)	21.7 (29.1)	29.2 (41.9)	0.752	34.3 (25.3)	29.2 (37.9)	0.848	13.5 (22.2)	0.006^*^
Whole-brain volume (L),^d^ median (IQR)	1.451 (0.17)	1.427 (0.15)	0.628	1.411 (0.14)	1.406 (0.14)	0.794	1.504 (0.16)	0.004*

CIS: clinically isolated syndrome; RRMS: relapsing-remitting multiple sclerosis; SPMS: secondary progressive multiple sclerosis; PPMS: primary progressive multiple sclerosis; EDSS: Expanded Disability Status Scale; DMT: disease-modifying therapy.

a For these group comparisons, the category “unknown” was not included.

b For this group comparison, CIS and RRMS were combined into one category.

c Disease duration represents the time between the first onset of neurological complaints and the visit date.

d An MRI scan was not available for five patients, of which one patient with poor performance validity.

e *p*-value represents the difference between three groups: cognitively impaired valid performers, cognitively impaired invalid performers, and cognitively preserved valid performers.

* Significantly different between groups. The cognitively preserved invalid performers are not presented, as this subgroup was too small (*N* = 2).

**Table 2.** Cognitive and patient-reported outcomes stratified by performance validity.

	Total sample (*N* = 99)					Classified as cognitively impaired (*N* = 62)					Classified as cognitively preserved (*N* = 37)		
	Valid performers (*N* = 79, 80%)	*N*	Invalid performers (*N* = 20, 20%)	*N*	*p*	Valid performers (*N* = 44, 71%)	*N*	Invalid performers (*N* = 18, 29%)	*N*	*p*	Valid performers (*N* = 35, 95%)	*N*	*p* ^d^
Performance validity													
ASTM	88.0 (2.0)^b^	79	78.0 (8.0)^b^	20	n/a	86.0 (4.0)^b^	44	77.0 (9.0)^b^	18	n/a	88.0 (2.0)^b^	35	n/a
Cognitive function													
Cognitively impaired	44 (56%)^c^	79	18 (90%)^c^	20	0.004*	n/a		n/a			n/a		
Overall cognition	−1.0 (0.8)^a^	79	−1.8 (1.0)^a^	20	0.001*	−1.6 (0.8)^b^	44	−1.6 (1.3)^b^	18	0.114	−0.4 (0.5)^b^	35	n/a
Processing speed	−1.2 (1.1)^a^	79	−1.7 (1.1)^a^	20	0.078	−1.7 (1.1)^a^	44	−1.8 (1.1)^a^	18	0.722	−0.6 (0.7)^a^	35	n/a
Memory—verbal	−0.8 (1.1)^a^	79	−1.9 (1.4)^a^	20	<0.001*	−1.3 (1.1)^b^	44	−1.9 (1.9)^b^	18	0.085	0.1 (1.0)^b^	35	n/a
Memory—visuospatial	−0.9 (1.5)^b^	72	−1.3 (2.8)^b^	16	0.294	−1.6 (1.2)^b^	38	−1.5 (2.6)^b^	14	0.951	−0.2 (0.9)^b^	34	n/a
EF—verbal fluency	−1.0 (0.9)^b^	77	−0.9 (1.6)^b^	14	0.754	−1.1 (0.8)^b^	43	−1.1 (1.3)^b^	12	0.919	−0.9 (1.2)^b^	34	n/a
EF—response inhibition	−1.4 (1.3)^a^	78	−2.4 (1.4)^a^	20	0.004*	−1.9 (1.5)^b^	43	−2.0 (1.9)^b^	18	0.179	−0.7 (1.2)^b^	35	n/a
Patient-reported outcomes													
Cognitive complaints	33.2 (9.3)^a^	70	35.4 (8.7)^a^	19	0.344	35.0 (10.0)^b^	40	34.0 (8.0)^b^	17	0.767	31.0 (12.0)^b^	30	0.728
Anxiety	8.6 (4.4)^a^	78	8.8 (4.4)^a^	20	0.876	8.0 (6.0)^b^	43	8.5 (6.5)^b^	18	0.745	8.0 (4.0)^b^	35	0.850
Depression	7.0 (4.2)^a^	78	8.0 (4.4)^a^	20	0.381	8.0 (6.0)^b^	43	7.0 (5.0)^b^	18	0.793	6.0 (5.0)^b^	35	0.160
Fatigue	39.1 (10.8)^a^	77	43.4 (9.9)^a^	20	0.111	40.4 (9.9)^a^	43	43.5 (10.5)^a^	18	0.282	37.4 (11.8)^a^	34	0.145
Sleep disturbances	7.7 (4.7)^a^	79	8.7 (4.6)^a^	20	0.367	8.3 (4.6)^a^	44	8.50 (4.8)^a^	18	0.903	6.8 (4.7)^a^	35	0.261
Quality of life													
Physical	50.9 (20.7)^b^	65	39.8 (23.4)^b^	17	0.303	42.9 (23.4)^b^	33	39.5 (25.3)^b^	15	0.911	55.9 (17.9)^b^	32	0.025
Mental	51.5 (30.4)^b^	67	53.3 (33.5)^b^	16	0.552	47.4 (28.3)^b^	36	61.1 (34.4)^b^	14	0.469	65.1 (35.9)^b^	31	0.171
Pain (subscale)	66.7 (46.7)^b^	75	63.3 (55.0)^b^	19	0.213	65.0 (45.8)^b^	42	55.0 (62.5)^b^	17	0.249	70.0 (50.0)^b^	33	0.408
Coping													
Active problem solving	18.0 (4.6)^a^	76	16.7 (3.4)^a^	18	0.247	17.5 (4.6)^a^	42	16.7 (3.5)^a^	16	0.512	18.6 (4.6)^a^	34	0.318
Palliative reaction	17.9 (3.6)^a^	74	17.9 (4.8)^a^	17	0.973	17.1 (3.1)^a^	40	17.7 (4.9)^a^	15	0.628	18.8 (4.0)^a^	34	0.162
Avoidance	17.0 (4.0)^b^	74	17.0 (8.0)^b^	14	0.819	17.0 (4.0)^b^	39	17.0 (7.0)^b^	12	0.763	17.0 (5.0)^b^	35	0.780
Seeking social support	13.5 (3.7)^a^	78	12.9 (3.7)^a^	18	0.556	13.1 (3.2)^a^	43	12.8 (3.8)^a^	16	0.711	14.0 (4.2)^a^	35	0.439
Passive reaction pattern	13.0 (4.0)^b^	77	13.0 (6.0)^b^	17	0.409	13.0 (3.0)^b^	43	13.0 (4.0)^b^	15	0.943	13.0 (5.0)^b^	34	0.877
Expressing emotions	7.0 (2.0)^b^	76	6.5 (4.0)^b^	18	0.619	7.0 (2.0)^b^	41	6.0 (3.0)^b^	16	0.836	6.0 (2.0)^b^	35	0.970
Reassuring thoughts	13.0 (3.0)^b^	76	11.0 (4.0)^b^	17	0.173	12.0 (3.0)^b^	41	12.0 (5.0)^b^	15	0.628	13.0 (4.0)^b^	35	0.218

ASTM: Amsterdam Short-Term Memory; EF: executive functioning; n/a: not applicable.

a Mean value (SD).

b Median (interquartile range).

c *n* (%).

d *p*-value represents the difference between three groups: cognitively impaired valid performers, cognitively impaired invalid performers, and cognitively preserved valid performers. The cognitively preserved invalid performers are not presented, as this subgroup was too small (*N* = 2). Cognitive scores represent the *z*-scores corrected for age, education, and sex.

When applicable, *p*-values of 0.05 were divided by the number of subscales, and were thereby set at *p* < 0.01 for cognitive (sub-)domains, *p* < 0.007 for coping style, *p* < 0.017 for quality of life subscales, and *p* < 0.025 for depression and anxiety.

* Significantly different between groups.

**Figure 2.**
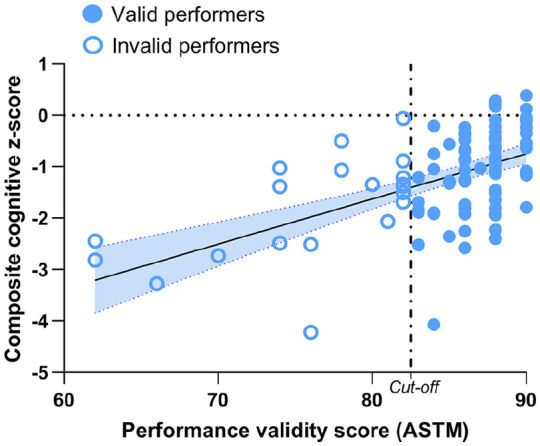
Regression analysis between performance validity and cognitive functioning. The regression analysis was calculated within the total MS sample, and the figure also illustrates which observations belonged to which performance validity groups. The cut-off score of the ASTM was set at ⩽82 to categorize invalid and valid performance. ASTM: Amsterdam Short-Term Memory.

In the total sample, the regression analysis showed that lower PVT scores (i.e. tending toward lower validity) related to lower overall cognitive functioning (β = 0.55, *p* < 0.001; Figure 2), and PVT scores explained 29% of the variance in overall cognitive functioning. Lower PVT scores correlated with lower processing speed (*p* = 0.002), verbal memory (*p* < 0.001), visuospatial memory (*p* < 0.001), and response inhibition (*p* < 0.001) scores, as well as higher EDSS scores (*p* = 0.021), the male sex (*p* = 0.008), and a lower education (*p* = 0.008). The other variables, including patient-reported outcomes, were not significantly related to PVT scores (Table 3).

**Table 3.** Correlations between performance validity scores and demographic, disease-related, cognitive, and patient-reported outcomes within the total sample.

	ASTM	
	Spearman’s *r*	*p*
Demographics		
Age (years)	−0.09	0.359
Sex (women)	0.27	0.008*
Education (high)	0.26	0.008*
Disease-related characteristics		
Disease duration (years)^a^	0.08	0.417
EDSS	−0.23	0.021*
Lesion load (mL)	−0.15	0.163
Whole-brain volume (L)	0.18	0.076
DMT use (yes)	−0.01	0.921
Cognitive function		
Overall cognitive functioning	0.53	<0.001*
Processing speed	0.31	0.002*
Memory—verbal	0.44	<0.001*
Memory—visuospatial	0.41	<0.001*
EF—verbal fluency	0.16	0.130
EF—response inhibition	0.39	<0.001*
Patient-reported outcomes		
Cognitive complaints	−0.03	0.795
Anxiety	0.01	0.942
Depression	−0.09	0.358
Fatigue	−0.11	0.289
Sleep disturbances	−0.15	0.152
Quality of life		
Physical	0.21	0.054
Mental	0.06	0.598
Pain	0.14	0.166
Coping		
Active problem solving	0.14	0.184
Palliative reaction	0.10	0.359
Avoidance	−0.003	0.981
Seeking social support	0.19	0.069
Passive reaction pattern	−0.09	0.416
Expressing emotions	0.03	0.807
Reassuring thoughts	0.20	0.058

ASTM: Amsterdam Short Term Memory; EDSS: Expanded Disability Status Scale; DMT: disease-modifying therapy; EF: executive functioning.

a Disease duration represents the time between the first onset of neurological complaints and the visit date.

*p*-values of 0.05 were divided by the number of subscales, and were thereby set at *p* < 0.01 for cognitive (sub-)domains, *p* < 0.007 for coping style, *p* < 0.017 for quality of life subscales, and *p* < 0.025 for depression and anxiety.

* All other correlations were set at *p*<.05. *significantly related to PVT scores.

Table 4 shows the concordance between the embedded PVTs and the ASTM. The percentage of patients categorized into the same validity category (i.e. concordance rates) varied between 73% and 85%.

**Table 4.** Concordance between the stand-alone performance validity test and embedded performance validity indices.

Embedded PVTs^25,26^	Stand-alone PVT (ASTM)		
CVLT-II discrimination variability	Valid	Invalid	Concordance rate
>80—valid	74/99 (75%)	14/99 (14%)	81%
⩽80—invalid	5/99 (5%)	6/99 (6%)	
BVMT-R discrimination variability	Valid	Invalid	Concordance rate
>3—valid	71/86 (83%)	12/86 (14%)	85%
⩽3—invalid	1/86 (1%)	2/86 (2%)	
Stroop word naming	Valid	Invalid	Concordance rate
>66s—valid	72/98 (73%)	17/98 (17%)	76%
⩾66s—invalid	6/98 (6%)	3/98 (3%)	
Stroop color naming	Valid	Invalid	Concordance rate
<93s—valid	69/98 (70%)	17/98 (17%)	73%
⩾93s—invalid	9/98 (9%)	3/98 (3%)	
COWAT *t*-score	Valid	Invalid	Concordance rate
>32—valid	74/91 (81%)	12/91 (13%)	83%
⩽32—invalid	3/91 (3%)	2/91 (2%)	

PVT: performance validity test; ASTM: Amsterdam Short Term Memory test; CVLT-II: Dutch version of the California Verbal Learning Test Version 2; BVMT-R: Brief Visuospatial Memory Test–Revised; COWAT: Controlled Oral Word Association Test.

Concordance rate represents the percentage of patients categorized into the same validity category by the ASTM and the embedded PVT.

### Performance validity of cognitively impaired and cognitively preserved patients

Invalid performance was found in 29% (18/62) of patients with cognitive impairment. Cognitively impaired valid and invalid performers did not differ regarding demographic, disease-related, patient-reported, or cognitive outcomes (Tables 1 and 2). In addition, invalid performance was found in only 5% (2/37) of cognitively preserved patients (i.e. due to the small sample size, no statistical analyses were performed with this subgroup). Tables 1 and 2 present the characteristics of cognitively preserved valid performers (*N* = 35).

**Figure 3.**
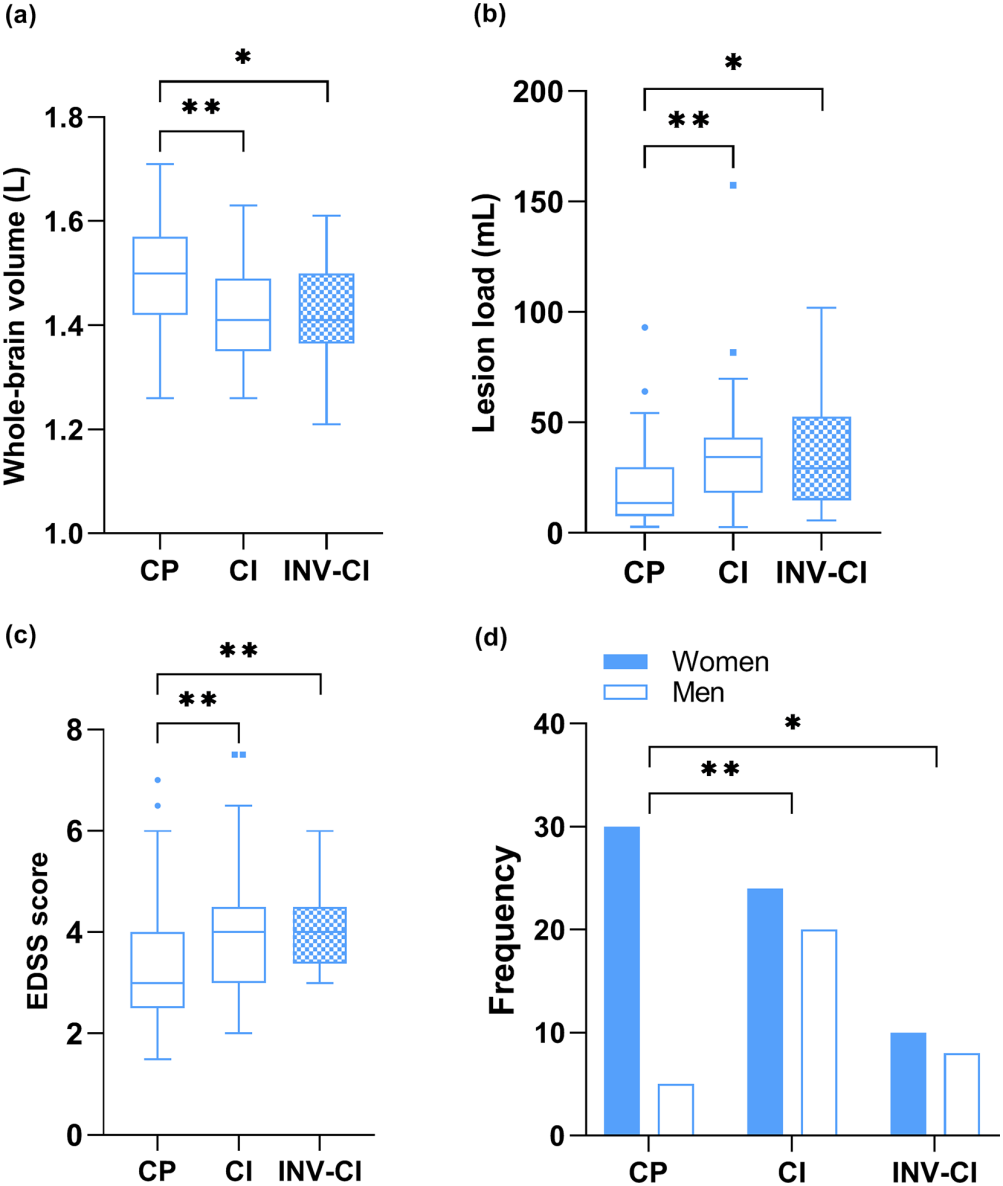
Group comparisons between cognitive and performance validity subgroups: (a) whole-brain volume per group, (b) lesion load per group, (c) Expanded Disability Status Scale (EDSS) score per group, and (d) frequency of men and women per group. CP: cognitively preserved valid performers; CI: cognitively impaired valid performers; INV-CI: invalid performers classified as cognitively impaired; EDSS: Expanded Disability Status Scale. *p < 0.05; **p < 0.01.

The subgroups (i.e. cognitively preserved valid performers, cognitively impaired valid performers, and cognitively impaired invalid performers) differed with regard to disease severity and sex (*p* < 0.05; Table 1 and Figure 3). Specifically, the cognitively impaired valid and invalid performers had a higher lesion load (*p* = 0.003 and *p* = 0.027, respectively), smaller whole-brain volume (*p* = 0.002 and *p* = 0.020, respectively), and higher disability level (*p* = 0.004 and *p* = 0.009, respectively) than the cognitively preserved valid performers, and these groups consisted of relatively more males than the cognitively preserved valid performers (*p* = 0.003 and *p* = 0.016, respectively).

## Discussion

This retrospective study investigated performance validity in a clinical cohort of MS patients with cognitive complaints, of which 20% had indications of suboptimal cognitive performance. This percentage corresponds to previous literature^7^ and may reflect the incidence of suboptimal performance in a clinical MS population with cognitive complaints. Notably, even in an MS study where cognitive functioning was not the primary outcome, 13% of the patients performed within the invalid range,^8^ indicating that suboptimal performance during neuropsychological evaluations also occurs in the general MS population.

MS patients with invalid performance had worse cognitive functioning compared to valid performers, and 90% of these patients would be classified as cognitively impaired if performance validity was not considered. In addition, performance validity scores accounted for 29% of the variance in cognitive functioning in our total sample. These results are not surprising as lower cognitive scores are expected among patients who perform suboptimally. Although PVTs do measure true cognitive abilities to a certain extent, the actual memory load of the ASTM (i.e. PVT used in this study) is minimal,^3^ and there were no indications that our MS sample was too severely affected in terms of cognitive abilities to pass a PVT. More specific, this study showed that the majority of patients classified as cognitively impaired adequately performed the PVT (71%), which was also reported in a previous MS study,^7^ and the invalid performers did not have lower cognitive scores than the cognitively impaired valid performers. These results indicate that PVT failure cannot be attributed to cognitive impairment itself.^7,33^ Importantly, PVT failure does not indicate intentionality, nor that patients are cognitively intact,^2^ but the substantial percentage found in this study does stress that clinicians should be aware of suboptimal performance in MS patients.

It is relevant to identify whether the severity of the disease may contribute to suboptimal performance in MS. Invalid and valid performers classified as cognitively impaired did not differ regarding disease status (e.g. disease duration and cerebral compromise), indicating that suboptimal performance is not a result of disease severity. However, cognitively impaired invalid performers were more severely affected regarding cerebral compromise and disability level than cognitively preserved valid performers. In addition, lower PVT scores were related to higher disability levels. Suboptimal performance thereby does *not* imply that patients are only mildly affected or exaggerating their symptoms. Instead, low cognitive scores should always be taken seriously. Even when patients perform suboptimally, they could still have actual cognitive impairments and suboptimal performance could be a way to (either consciously or unconsciously) express their disease burden. In general, regardless of the severity of their disease, if patients fail a PVT and perform within the cognitively impaired range, cognitive impairments cannot be confirmed nor ruled out. Note that in patients with profound cognitive impairments or who meet the criteria for major neurocognitive disorder (i.e. dementia), invalid performance on PVTs should be judged with caution because they are less reliable.^2,3^ Performance on conventional neuropsychological tests should therefore be interpreted using a clinical approach.

Suboptimal performance has mainly been linked to mood or external incentives in previous MS studies.^6,7^ Our patient sample visited the outpatient clinic for clinical purposes, without recognizable external incentives to perform poorly on a PVT, although external incentives cannot be completely ruled out. A larger percentage of patients with invalid performance received sickness benefits (40%) than the valid performers (14%), but this difference was not significant. Our results suggest that suboptimal performance cannot be explained by psychological burden, nor by fatigue or pain. In addition, the way patients cope with problems did not explain suboptimal performance. Potentially, suboptimal performance is induced by emotional or behavioral aspects not captured by standardized questionnaires, such as the need to have their cognitive complaints acknowledged,^34^ or to express feelings of distress.

We did find indications that demographic characteristics, including being male and lower education, related to worse PVT scores. Lower educated MS patients may have greater difficulty to perform a PVT than higher educated patients, although lower educated patients did not show a higher percentage of PVT failure in our sample. With regard to the male sex, there were relatively more males among both the valid and invalid performing cognitively impaired patients than among cognitively preserved patients. This could reflect that males are more severely affected than females, as our results do not specifically indicate that males more often fail a PVT. Further examination is needed to interpret the meaning of this relation, as these findings could also suggest that clinicians need to be more alert for suboptimal performance when evaluating cognitive performance of lower educated and male MS patients. Overall, it remains largely unpredictable which patients perform suboptimally during neuropsychological assessment. Potential underlying neurological and psychological mechanisms should therefore be further investigated, and qualitative interviews with patients who perform suboptimally may be crucial to gain a better understanding of suboptimal performance as quantitative measures may not capture the whole story.

There is no uniform guideline on how poor performance validity should subsequently be handled, but it has been suggested that feedback upon PVT failure may increase the likelihood of valid performance on re-testing in MS,^6^ although these results could not be confirmed in another clinical population.^35^ There is consensus that cognitive test results of patients who perform suboptimally cannot be validly interpreted, as this can lead to incorrect diagnoses and consequently unsuitable treatments.^35^ In addition, suboptimal performance may induce noise in research data which leads to difficulty in determining the mechanisms underlying cognitive impairment in MS.^35^ Thereby, including a PVT in a neuropsychological assessment allows for a more nuanced conclusion of the patients’ cognitive performance, and it may increase the chance of finding the underlying mechanisms of true cognitive impairments. In addition to including a stand-alone PVT (i.e. a test specifically designed to detect suboptimal performance), performance validity indicators within conventional neuropsychological tests (i.e. embedded PVTs) may provide additional information on suboptimal performance. However, we found low concordance between the stand-alone PVT and embedded PVTs when categorizing patients’ performance as invalid, which corresponds to a previous MS study^7^ and may be due to the low sensitivity levels of embedded PVTs.^25^ The value of embedded PVT indicators needs further exploration in MS.

This study had some limitations. We included only one stand-alone PVT to determine suboptimal test performance. Even though we applied a more conservative cut-off score to obtain a high specificity (95%), thereby reducing the chance of incorrectly categorizing patients’ performance as suboptimal, combining at least two PVTs may have led to more firm conclusions.^33^ In addition, PVTs have not yet been validated in MS, and it is relevant to investigate which combination of PVTs obtains the best psychometric properties in this population.^25^ The ASTM is a verbal memory-based PVT and future research could include PVTs that tap other cognitive abilities (e.g. information processing speed). Moreover, the group that failed a PVT was relatively small (*N* = 20), and it could be that larger patient groups do reveal psychological differences. Also, larger patient groups make it possible to examine the value of specific combinations of predictors, which may provide more insight into suboptimal performance. Finally, the PVT was performed at the end of the neuropsychological assessment. Even though overall fatigue was not related to PVT scores in our sample, it could be that fatigue-related complaints during neuropsychological testing influenced the outcomes.

In conclusion, our results indicate that suboptimal performance regularly occurs among MS patients with cognitive complaints. Even though it remains difficult to grasp the underlying reasons of suboptimal performance, absence of PVTs may result in invalid interpretations of cognitive test results and consequently in less relevant patient education and counseling. Performance validity during neuropsychological assessments of MS patients thereby warrants attention in clinical and research settings. No satisfactory explanation for suboptimal performance could yet be detected, and as such, future studies should investigate why PVT failure occurs in a substantial percentage of MS patients with cognitive complaints.

## Supplemental Material

sj-pdf-1-msj-10.1177_13524585211025780 – Supplemental material for Performance validity in outpatients with multiple sclerosis and cognitive complaints Click here for additional data file. Supplemental material, sj-pdf-1-msj-10.1177_13524585211025780 for Performance validity in outpatients with multiple sclerosis and cognitive complaints by IM Nauta, D Bertens, M van Dam, M Huiskamp, S Driessen, JJG Geurts, BMJ Uitdehaag, L Fasotti, HE Hulst, BA de Jong and M Klein in Multiple Sclerosis Journal

sj-pdf-2-msj-10.1177_13524585211025780 – Supplemental material for Performance validity in outpatients with multiple sclerosis and cognitive complaints Click here for additional data file. Supplemental material, sj-pdf-2-msj-10.1177_13524585211025780 for Performance validity in outpatients with multiple sclerosis and cognitive complaints by IM Nauta, D Bertens, M van Dam, M Huiskamp, S Driessen, JJG Geurts, BMJ Uitdehaag, L Fasotti, HE Hulst, BA de Jong and M Klein in Multiple Sclerosis Journal
