# Supplementary material for: Performance validity in outpatients with multiple sclerosis and cognitive complaints
Source: Mult Scler. 2021 Jul 2;28(4):642–53. doi: 10.1177/13524585211025780 (PMC8961248; doi:10.1177/13524585211025780)
Supplement: sj-pdf-2-msj-10.1177_13524585211025780 – Supplemental material for Performance validity in outpatients with multiple sclerosis and cognitive complaints [file sj-pdf-2-msj-10.1177_13524585211025780.pdf]

Supplementary Information - methods

Measures

Disease status

Patients were scanned on a 3-Tesla whole-body MRI (General Electric Signa HDxt), as described previously.[1] Lesion load and whole-brain volume were calculated as indicators of cerebral damage. A 3D fluid attenuated inversion recovery (FLAIR) sequence (repetition time: 8000 ms, echo time: 125 ms, inversion time: 2350 ms, slice thickness: 1.20 mm, in-plane resolution: 0.98x0.98mm) was acquired for white matter lesion detection. A 3D T1-weighted fast-spoiled gradient echo (repetition time: 7.8 ms, echo time: 3.0 ms, inversion time: 450 ms, flip angle: 12°, slice thickness: 1.20 mm, in-plane resolution: 0.90x0.90 mm) was acquired for the calculation of brain volumes.

White matter lesions were quantified by applying auto-segmentation (i.e. k-nearest neighbor classification) on the FLAIR and 3D T1 sequences.[2] Lesion load was calculated based on these segmentations. By using an automated lesion-filling technique (LEAP), lesions were filled on the 3D T1 sequence.[3] Next, whole-brain volume was computed with SIENAX,[4] using FSL (http://www.fmrib.ox.ac.uk/fsl). To correct lesion load and whole-brain volume for head size, the V-scaling factor derived by SIENAX was used, resulting in normalized volumes.

Neuropsychological examination

Cognitive function was measured with a test battery based on the MACFIMS.[5] Verbal memory was assessed with the Dutch version of the California Verbal learning Test version 2 (CVLT-II), and we used the total recall over all five trials, the long-delayed recall and recognition as outcome measures.[6] Visuospatial memory was assessed with the Brief Visuospatial Memory Test-Revised (BVMT-R) using the total score of the three trials, the delayed recall and delayed recognition.[7] Processing speed was measured with the Symbol Digit Modalities Test (SDMT), of which the total number of correctly substituted symbols within 90 seconds was registered,[8] and the Stroop Color-Word Test, using the time to complete cards I and II.[9] Within the executive function domain, verbal fluency was measured with the Controlled Oral Word Association Test (COWAT; total score of three letters),[10] and response inhibition with the interference score of the Stroop Color-Word Test (card III minus the mean of cards I and II).[9]

Consistent with Amato et al.[11] and previous publications from our group,[12] individual cognitive test scores were adjusted for the effects of age, sex and education when appropriate based on a normative sample of Dutch healthy controls. These corrected scores were subsequently converted into z-scores based on the same normative sample. Accordingly, these z-scores were transformed into (sub-)domain-specific z-scores (i.e. verbal memory, visuospatial memory, processing speed, verbal fluency, response inhibition) as well as one composite score for overall cognitive functioning (i.e. average of all z-scores) based on a normative sample of healthy controls.[11, 12] Higher z-scores represent better performance. Patients were classified as cognitively impaired (CI) if they scored at least 1.5 SDs below the mean of the healthy controls on at least 20% of the test scores (this corresponded to 3 deviant scores out of 11 test scores), as recommended in previous studies.[13, 14] Patients who did not fulfill these criteria were classified as cognitively preserved (CP).

Patient-reported outcomes

The Multiple Sclerosis Neuropsychological Questionnaire patient version (MSNQ-P) measured cognitive complaints.[15] The MSNQ-P consists of 15 items describing cognitive complaints, and scores range from 0 to 60. The Hospital Anxiety and Depression Scale measured symptoms of anxiety and depression.[16] Both scales contain seven items and scores on each scale range from 0 to 21. The subscale 'subjective experience of fatigue' of the Checklist Individual Strength-20-r assessed the level of fatigue.[17] This subscale consists of eight items and the total score ranges from 0 to 56. The Athens Insomnia Scale measured sleep disturbances.[18] This questionnaire consists of eight items and scores range between 0 and 24. For all of the aforementioned questionnaires, higher scores indicate more symptoms. The Utrecht Coping List measured the patients' coping style, which consists of the subscales active problem solving (seven items; scores range 0-28), palliative reaction (eight items; scores range 0-32), avoidance (eight items; scores range 0-32), seeking social support (six items; scores range 0-24), passive reaction pattern (seven items; scores range 0-28), expressing emotions (three items; scores range 0-12), and reassuring thoughts (five items; scores range 0-20).[19] Subscales were analyzed separately. Higher scores indicate that a patient predominantly adopts a specific coping style. The MS Quality of Life Questionnaire-54 measured physical and mental quality of life. The physical and mental quality of life indexes, which consist of eight and five subscales respectively, were

included as well as the pain subscale. Scores on all three outcomes could range between 0 and 100, and higher scores represent better quality of life.[20]

References

1. van Geest Q, Douw L, van't Klooster S, et al. Information processing speed in multiple sclerosis: Relevance of default mode network dynamics. *Neuroimage-Clin* 2018; 19: 507-515. DOI: 10.1016/j.nicl.2018.05.015.

2. Steenwijk MD, Pouwels PJ, Daams M, et al. Accurate white matter lesion segmentation by k nearest neighbor classification with tissue type priors (kNN-TTPs). *Neuroimage Clin* 2013; 3: 462-469. DOI: 10.1016/j.nicl.2013.10.003.

3. Chard DT, Jackson JS, Miller DH, et al. Reducing the impact of white matter lesions on automated measures of brain gray and white matter volumes. *J Magn Reson Imaging* 2010; 32: 223-228. DOI: 10.1002/jmri.22214.

4. Smith SM, Zhang Y, Jenkinson M, et al. Accurate, robust, and automated longitudinal and cross-sectional brain change analysis. *Neuroimage* 2002; 17: 479-489.

5. Benedict RH, Cookfair D, Gavett R, et al. Validity of the minimal assessment of cognitive function in multiple sclerosis (MACFIMS). *J Int Neuropsychol Soc* 2006; 12: 549-558.

6. Mulder JL, Dekker R and Dekker DH. *Verbale Leer- & Geheugen test: Handleiding [Verbal Learning & Memory Test: Manual].* Lisse, the Netherlands: Swets & Zeitlinger, 1996.

7. Benedict RHB, Schretlen D, Groninger L, et al. Revision of the brief visuospatial memory test: Studies of normal performance, reliability, and validity. *Psychol Assessment* 1996; 8: 145-153. DOI: Doi 10.1037/1040-3590.8.2.145.

8. Smith A. *Symbol digit modality test (SDMT): manual (revised)*. Los Angeles: Psychological Services, 1982.

9. Hammes JGW. *The STROOP color-word test: Manual*. Amsterdam: Swets and Zeitlinger, 1973.

10. Benton LA, Hamsher KD and Sivan AB. Controlled Oral Word Association Test. *Multilingual aphasia examination*. 3 ed. Iowa City, IA: AJA, 1994.

11. Amato MP, Portaccio E, Goretti B, et al. The Rao's Brief Repeatable Battery and Stroop Test: normative values with age, education and gender corrections in an Italian population. *Mult Scler* 2006; 12: 787-793. DOI: 10.1177/1352458506070933.

12. Eijlers AJC, van Geest Q, Dekker I, et al. Predicting cognitive decline in multiple sclerosis: a 5-year follow-up study. *Brain* 2018; 141: 2605-2618.

13. Benedict RHB. Standards for sample composition and impairment classification in neuropsychological studies of multiple sclerosis. *Multiple Sclerosis* 2009; 15: 777-778. DOI: 10.1177/1352458509106713.

14. Fischer M, Kunkel A, Bublak P, et al. How reliable is the classification of cognitive impairment across different criteria in early and late stages of multiple sclerosis? *Journal of the Neurological Sciences* 2014; 343: 91-99. DOI: 10.1016/j.jns.2014.05.042.

15. Benedict RH, Munschauer F, Linn R, et al. Screening for multiple sclerosis cognitive impairment using a self-administered 15-item questionnaire. *Mult Scler* 2003; 9: 95-101.

16. Zigmond AS and Snaith RP. The Hospital Anxiety and Depression Scale. *Acta Psychiat Scand* 1983; 67: 361-370. DOI: DOI 10.1111/j.1600-0447.1983.tb09716.x.

17. Vercoulen JHMM, Alberts M and Bleijenberg G. De checklist individuele spankracht (CIS). *Gedragstherapie* 1999; 32: 131-136.

18. Soldatos CR, Dikeos DG and Paparrigopoulos TJ. Athens Insomnia Scale: validation of an instrument based on ICD-10 criteria. *Journal of Psychosomatic Research* 2000; 48: 555-560. DOI: Doi 10.1016/S0022-3999(00)00095-7.

19. Schreurs PJG, Van de Willige, G., Brosschot, J. F., Tellegen, B., & Graus, G. M. H. . *De Utrechtse Copinglijst: UCL. [The Utrecht Coping List: UCL].* . Lisse: Swets en Zeitlinger, 1993.

20. Vickrey BG, Hays RD, Harooni R, et al. A Health-Related Quality-of-Life Measure for Multiple-Sclerosis. *Qual Life Res* 1995; 4: 187-206. DOI: Doi 10.1007/Bf02260859.
